# DeepPep: Deep proteome inference from peptide profiles

**DOI:** 10.1371/journal.pcbi.1005661

**Published:** 2017-09-05

**Authors:** Minseung Kim, Ameen Eetemadi, Ilias Tagkopoulos

**Affiliations:** 1 Department of Computer Science, University of California, Davis, Davis, California, United States of America; 2 Genome Center, University of California, Davis, Davis, California, United States of America; Indiana University, UNITED STATES

## Abstract

Protein inference, the identification of the protein set that is the origin of a given peptide profile, is a fundamental challenge in proteomics. We present DeepPep, a deep-convolutional neural network framework that predicts the protein set from a proteomics mixture, given the sequence universe of possible proteins and a target peptide profile. In its core, DeepPep quantifies the change in probabilistic score of peptide-spectrum matches in the presence or absence of a specific protein, hence selecting as candidate proteins with the largest impact to the peptide profile. Application of the method across datasets argues for its competitive predictive ability (AUC of 0.80±0.18, AUPR of 0.84±0.28) in inferring proteins without need of peptide detectability on which the most competitive methods rely. We find that the convolutional neural network architecture outperforms the traditional artificial neural network architectures without convolution layers in protein inference. We expect that similar deep learning architectures that allow learning nonlinear patterns can be further extended to problems in metagenome profiling and cell type inference. The source code of DeepPep and the benchmark datasets used in this study are available at https://deeppep.github.io/DeepPep/.

## Author Summary

The accurate identification of proteins in a proteomics sample, called the protein inference problem, is a fundamental challenge in biomedical sciences. Current approaches are based on applications of traditional neural networks, linear optimization and Bayesian techniques. We here present DeepPep, a deep-convolutional neural network framework that predicts the protein set from a standard proteomics mixture, given all protein sequences and a peptide profile. Comparison to leading methods shows that DeepPep has most robust performance with various instruments and datasets. Our results provide evidence that using sequence-level location information of a peptide in the context of proteome sequence can result in more accurate and robust protein inference. We conclude that Deep Learning on protein sequence leads to superior platforms for protein inference that can be further refined with additional features and extended for far reaching applications.

This is a *PLOS Computational Biology* Methods paper.

## Introduction

The accurate identification of proteins in a proteomics sample is a key challenge in life sciences. Proteins, the final gene product and the fundamental blocks of all cellular processes, are elusive to detect. The standard technology that provides fast, high-throughput characterization of complex protein mixtures is mass spectrometry-based shotgun proteomics. Initially, proteins are fragmented in small amino acid chains that are called peptides that then pass through a mass spectrometer. This results in a specific mass spectrum signature for each peptide, which correlates current intensity with a peptide’s weight and charge. Next, this signature is matched to a peptide database to identify which peptides are present in the sample (peptide identification step). Finally, the peptide profile is used to predict which proteins were more likely to produce the observed peptide set (protein inference step) [1]. More precisely, the challenge in protein inference is to infer the proteins (output) that give rise to the peptides observed in the sample. Each peptide has been identified after a database search of the sample mass spectrum, with a certain confidence level, also known as the “peptide probability” [2].

The protein inference problem has been particularly challenging due to existence of degenerate peptides and ‘one-hit wonders’ [1]. A degenerate peptide is one that can be generated by multiple proteins. A ‘one-hit wonder’ is a protein that has only one peptide match. The tools that have been developed for tackling these challenges use a wide arsenal of algorithmic methods, including optimization and parsimonious techniques [3], non-parametric [4] and parametric models [5] (in particular, ensemble of machine-learning methods [6]), among others [7–9] (see [1] for an extensive review). Most recent advances [8, 9] rely on the quantification of peptide detectability, a measure of the detection probability for a given peptide from a standard sample mixture by a standard proteomics routine given its parent protein. That is, the peptide detectability can be considered as an inherent characteristic of a peptide that is primarily determined by its sequence and its parent protein sequence [1]. Such information is typically predicted from peptide features such as amino acid composition, N- and C- terminal residues. An intrinsic problem in estimating peptide detectabilities is the existence of different biological conditions, the unequal concentrations of proteins before preparation of the sample and the existence of errors, all of which complicate protein inference and introduce noise [7].

To address these challenging aspects of protein inference, we have developed a deep learning technique, DeepPep, that uses the sequence information of proteins and peptides as inputs. Artificial neural networks have been applied in the past in proteomics, with applications such as the prediction of the retention time in LC-MS data [10, 11], prediction of peptide detectability [7, 12, 13], prediction of peak intensity in a MS/MS spectrum [14] and more recently, prediction of protein secondary structure [15]. Notably, [13] used ensemble of 30 neural networks with one hidden layer where the number of hidden nodes ranges from 1 to 4 for prediction of peptide detectability and protein quantity. In a recent work, a neural network with one hidden layer is trained to differentiate target proteins from decoy proteins in target decoy dataset [16]. DeepPep differs from previous work as it uses convolution layers for capturing the sequence information of proteins and peptides, hence allowing for more complex nonlinear relationships. This introduces new computational challenges that we solve using advanced techniques ranging from regularization, efficient optimization method **Section 2.5**, and sparsity-aware, redundancy-aware computations **Section 2.7**. In the following sections we present the DeepPep’s architecture, the inference algorithm and its application in seven benchmark datasets.

## Methods

### Method overview

**Fig 1:**
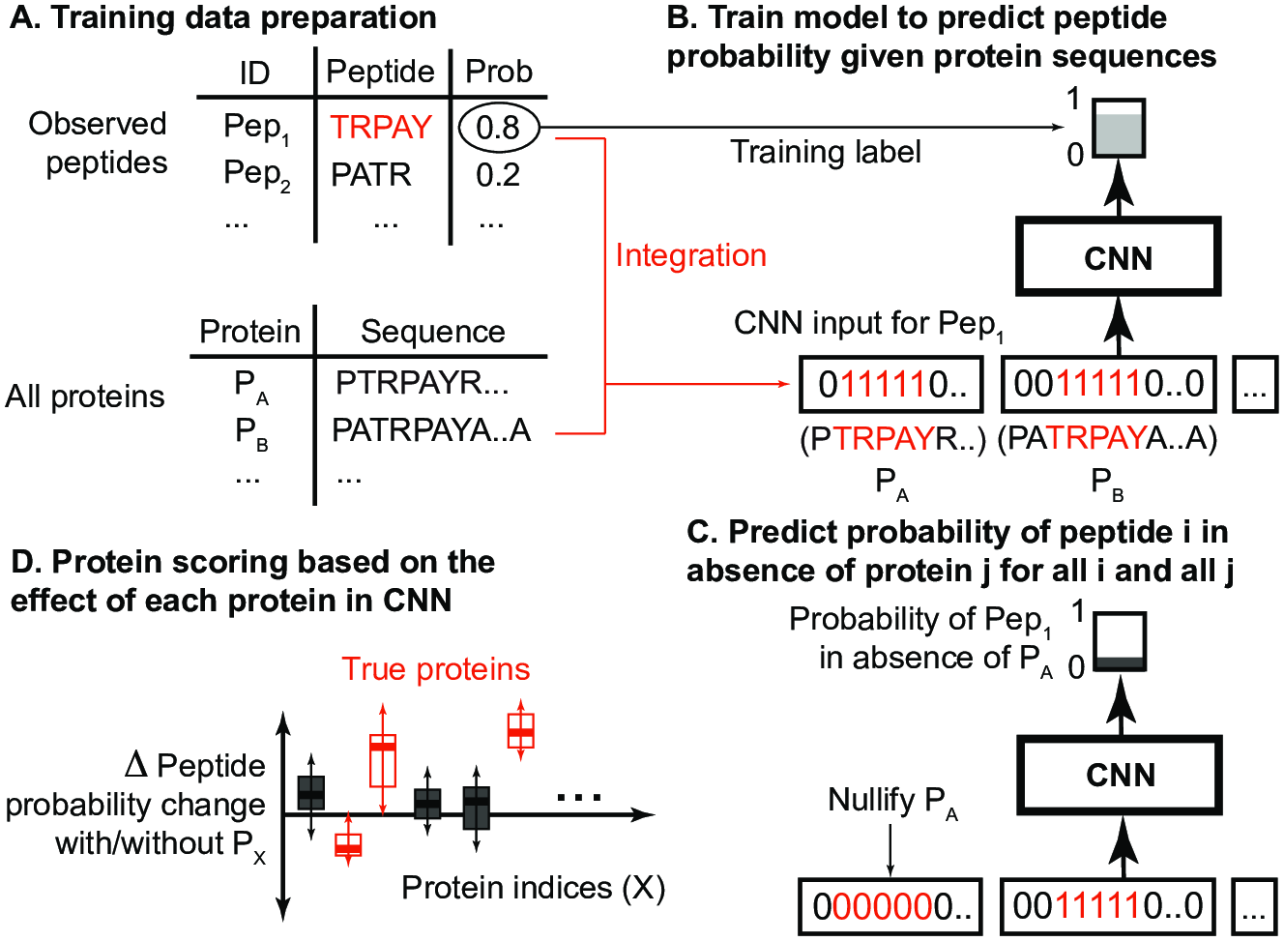
DeepPep overview. DeepPep takes as an input a set of strings for sequences of all the protein matches to an observed peptide. (A) To train the model for a specific peptide, each protein sequence string is converted to binary with ones where the peptide sequence matches that of the protein sequence, and zero everywhere else. (B) A CNN is then trained to predict the peptide probability. A peptide probability is the probability that the peptide that is identified through a database search from the mass spectra is the correct one. (C) The effect of a protein removal to a peptide probability is then calculated for all proteins and all peptides. (D) Finally, we score proteins based on differential change of each protein in CNN when it is present/absent.

DeepPep is a deep learning-based framework that predicts the list of scored proteins (output) that are more likely to generate a given peptide profile (input). Each candidate protein is scored based on its effect on peptide probability predictions of the deep learning model when it is present/absent **Section 2.6**. The framework is composed of four sequential steps as shown in Fig 1. The training data consist of two components: (a) the observed peptide sequences with associated probabilities returned by mass spectra database search [17], and (b) the whole set of proteins (and their sequences) for the respective organism. This data are imported to the convolutional neural network (CNN) for identifying complex patterns between the probability of observed peptides and the positional information of the peptide in the protein sequences.

There are two key ideas here that are translated to tasks for the CNN to (indirectly) learn: first, that a protein that matches multiple peptides in the sample is more likely to be part of the protein set that gave rise to that particular sample; second, a protein with non-overlapping peptide hits is more likely to exist in the sample than a protein with the same amount but overlapping peptide hits. “Non-overlapping peptide hits” are the peptide matches to a specific protein that do not coincide, that is, there is no position in the protein sequence where two or more peptides match. The more frequent occurrence of non-overlapping hits stems from the chemical treatment of the proteins; proteins are typically degraded to peptides by trypsin, which deterministically cleaves at the carboxyl side of the amino acids lysine or arginine (except when either is followed by proline [18]) and therefore, this deterministic process in protein degradation gives rise to non-overlapping peptides. In the next step, all peptide probabilities are predicted with and without the presence of each protein. We finally score the proteins based on the difference in peptide probability between being present and absent. This differential ranking is a key concept behind DeepPep. Conceptual basis of this idea is rooted from feature selection in machine learning [19], which is performed to find informative attributes in a prediction task. Protein inference methods using feature selection can be vulnerable to cases where attributes contain similar information. Although this can make such methods give low or zero weights to proteins containing homologous peptides, we observe that such cases are very limited in real datasets. For seven separate proteomic datasets (**Section 2.2**), the population of protein pairs having similar peptide matches (PCC > 0.7) represent on average below 0.05% among all possible pairwise comparisons of candidate proteins (**S1 Table** in S1 Text).

### Datasets

We used seven separate MS/MS datasets for evaluation purpose as follows:

- Sigma49: synthetic mixture of 49 proteins from Sigma Aldrich, made available by [8].
- UPS2: sample mixture consisting of 48 human proteins with various concentrations made available by [20].
- 18Mix: synthetic highly purified extract of 18 proteins [21].
- Yeast: yeast cellular extract with reference generated by intersection of various MS and non-MS based techniques [22].
- DME: protein extract of Drosophila melanogaster S2 cells [23].
- HumanMD: protein extract of human medulloblastoma Daoy cells [24].
- HumanEKC: protein extract of human embryonic kidney cells T293 [25].

The summary statistics and source information of the seven datasets are shown in **S2 Table** (in S1 Text). Four datasets of Sigma49, UPS2, 18Mix, and Yeast were the ones available with information of the true protein set that gave rise to the respective peptide profiles. For three remaining datasets (DME, HumanMD, HumanEKC) however the true protein set is unknown. To mitigate, we used the target decoy strategy [26] for evaluation in such datasets instead. A target decoy strategy adds a set of incorrect (i.e. decoy) proteins to the search space to be considered as true negatives during evaluation. More specifically we used the default decoy strategy in Trans Proteomic Pipeline (TPP v5.0.0) [27], which performs random shuffling of tryptic peptides of a real protein from database to generate a new decoy protein. For each dataset, given all available mass spectrometry files and the related reference protein database, peptide probabilities are calculated using TPP. For database search and for estimation of peptide identification probabilities, X!Tandem and PeptideProphet are used, respectively. The peptide detectabilities (as needed for running MSBayesPro and ProteinLasso) are estimated using a pre-trained model provided by DQmodel [7] team. For optimizing hyper-parameters of Fido, we used the target decoy strategy over each dataset where the performance is measured based on how well the method differentiates target proteins from decoy proteins without looking into the true protein set. This doubles the size of each search dataset by adding decoy protein sequences for the amount of target proteins. For investigation of hyper-parameters for DeepPep, we employ the same approach used for Fido but only on 18Mix dataset. We would like to note that this strategy doesn’t use the information of the true protein set that gives rise to the observed profiles and thus can be used for evaluation purpose with regards to prediction of the true proteins.

### Data preparation

The training dataset consists of training pairs {*x*_*i*_, *y*_*i*_} for each peptide *pp*_*i*_. The actual number of peptides that are present in any proteomics profile differ and can be from a few hundred to thousands. The input *x*_*i*_ is a set of binary vectors that has been constructed by (a) first preparing the amino-acid sequences of all proteins that have at least one match for any of the observed peptides in the profile, (b) then replacing their amino-acid sequences of each protein with ones in any place where there is a full match of peptide *pp*_*i*_ and zeros otherwise. In other words, *x*_*i*_ is a set of vectors that contains the location where the peptide *pp*_*i*_ matches to the organism’s proteome. The input *y*_*i*_ is the probabilistic score of a specific peptide-spectrum match for peptide *pp*_*i*_, calculated from PeptideProphet [2]. In other words, it is the identification probability of a peptide in mass spectra returned by a database search. We choose the highest probability in case there exist multiple spectra matches to the same peptide. The construction of input sequence (*x*_*i*_) is described in **Algorithm 1** and it has to be applied for each of the observed peptides.

**Algorithm 1** Build CNN Training Data

**Comment:** $\hat{p}$ is the set of sequences for the candidate proteins *p*. ${\hat{pp}}_{i}$ is the sequence of peptide *pp*_*i*_. *x*_*j*_[*m*, *n*] is the output string from position *m* to *n* for protein *p*_*j*_.

**Input:** $\hat{p}$, $\hat{pp_{i}}$

**Output:** *x*

**for** *j* = 1 **to** |*p*| **do**

 Initialize *x*_*j*_ to zeros where $|x_{j}\left|=\right|{\hat{p}}_{j}|$

 **for** *m* = 1 **to** $|{\hat{p}}_{j}\left|-\right|{\hat{pp}}_{i}|+1$ **do**

   **if** $\text{equal}({\hat{p}}_{j}\left[m,m+\right|{\hat{pp}}_{i}|-1],{\hat{pp}}_{i})$ **then**

    $x_{j}\left[m,m+\right|{\hat{pp}}_{i}\left|-1\right]=1$

   **end if**

  **end for**

**end for**

### CNN architecture

**Fig 2:**
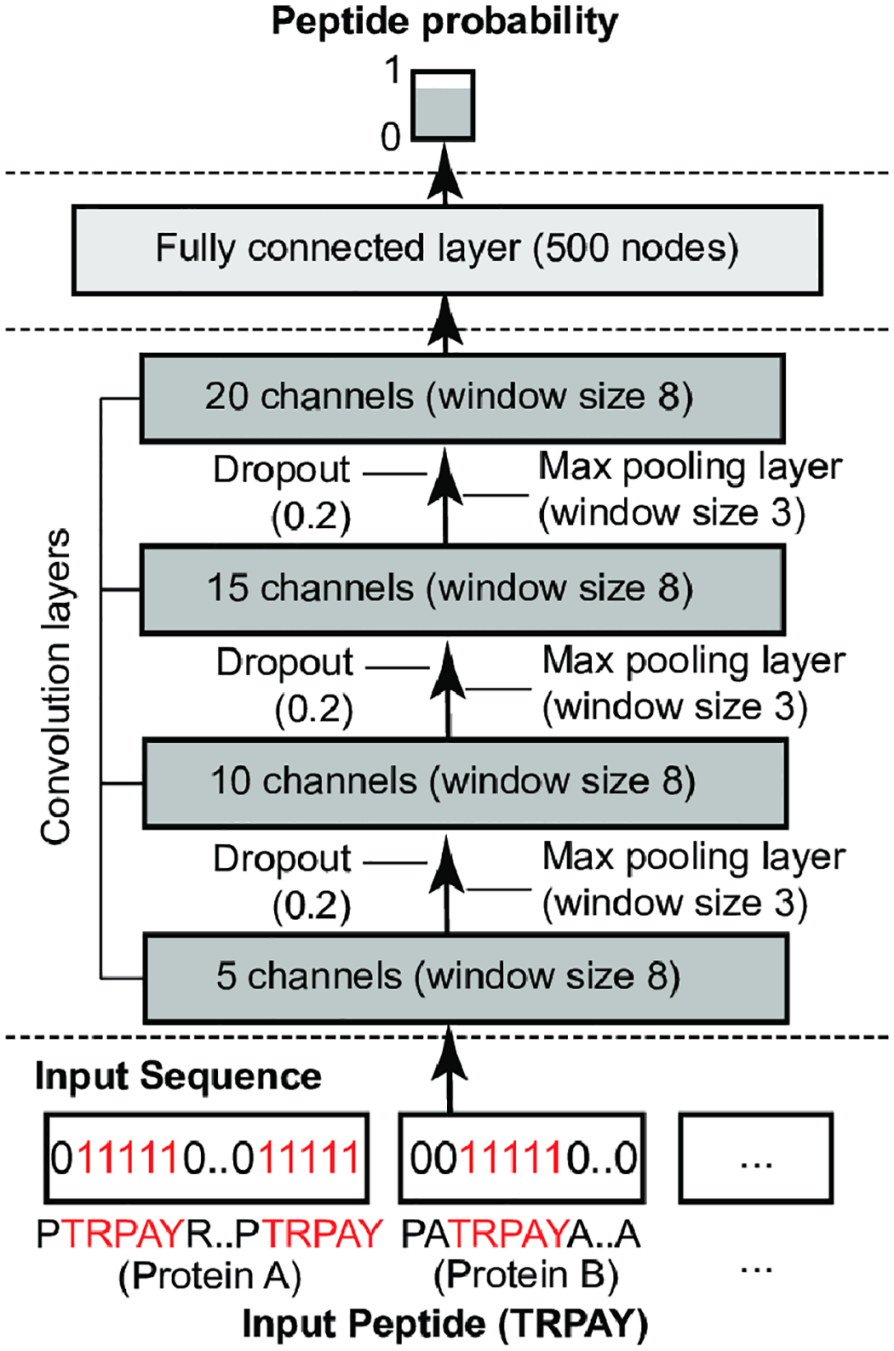
The architecture of CNN. Input peptide constructs the n set of input sequences where n is the number of all proteins and the position of the input peptide is marked in binary mode. For example, the input peptide (TRPAY) marks ones where it has matches in each of protein sequences, otherwise zeros. The input sequence is processed in four sequential convolution layers where a pooling layer and dropout are applied between each. Fully connected layer is applied after the fourth convolution layer, which produces the output value of predicted peptide probability. ReLU function was used for all transformations.

The deep convolutional network (CNN) is organized by a series of layers (Fig 2). Unlike typical CNNs, the CNN in the DeepPep framework has distinctive binary vectors as its input layer to represent sequences of proteins encoding input peptides. The organization of layers in the CNN architecture is similar to what has been used before in other fields [28], alternating a convolution layer and a pooling layer four times, followed by a fully connected layer and finally overlaying an output layer that predicts the peptide probability. Each layer computes a linear transformation of the output from the previous layer multiplied by a weight matrix, followed by a nonlinear transformation (ReLU). The only exception is at output layer where it produces linear value from the previous layer without applying the transformation.

The CNN uses three different types of layers: the convolution layer, the pooling layer and the fully connected layer. The convolution layer computes output by one-dimensional convolution operation with a specified number of filters (weight matrices) and all convolution operation outputs are then transformed by the rectified linear activation function (ReLU):

$$X_{i,f}^{p,l}=\text{ReLU}\left(\sum_{j=1}^{w}\;\sum_{k=1}^{n}\;W_{j,k}^{f,l-1}X_{i+j,k}^{p,l-1}\right)$$

where *X* is the input, *p* is the index of protein, *l* is the index of the convolution layer, *i* is the index of the output position and *f* is the index of filters. Each convolution filter *W*^*f*,*l*^ is an *w* × *n* weight matrix of filter *f* at layer *l* with *w* being the window size and *n* being the number of input channels.

A pooling layer computes the function *pool* (max-pooling) in a window with the specified length (*w*) for each filters computed from preceding convolutional layers. The step size is equal to the size of the pooling window. This reduces the size of the output to that of the window size and thus allows sequence learning patterns of higher abstraction in the next convolution layer:

$$X_{i,f}^{p,l}=\text{pool}\left(\left\{X_{iw,f}^{p,l-1},X_{iw+1,f}^{p,l-1}...X_{iw+w-1,f}^{p,l-1}\right\}\right)$$

where *X*^*p*,*l*^ is the input of protein *p* from preceding convolution layer *l* − 1, *i* is index of output position, *f* is index of filter, *w* is pooling window size.

The number of filters for each of the four convolution layers increase at each level (5, 10, 15 and 20 respectively), so that higher-level convolution layers can produce more complex patterns. We added a fully connected layer over the fourth pooling layer, which is the high-level representation of information computed from the binary proteome sequence that encodes the input peptide profile. This fully connected layer computes the product *W*^*l*^*X*^*l*^, where *X*^*l*^ is the combined input of all proteins from convolution layer *l* and *W*^*l*^ is the weight matrix for the fully connected layer. There is one weight matrix *W*^*l*^ corresponding to the output node of the output layer that calculates the predicted peptide probability. The optimal configuration with respect to the pooling function *pool*, number of filters, window sizes in convolution layer and pooling layer and number of nodes in fully connected layer was empirically determined (**Section**) and the final architecture is depicted in Fig 2.

### Training of CNN

The objective function is the minimization of the sum of squared errors between predicted and measured peptide probability:

$$\text{minimize}\;\sum_{i=1}\;{\left(y_{i}-{\hat{y}}_{i}\right)}^{2}$$

where *i* is the peptide index of all observed peptides and *y*_*i*_ indicates the measured probability of peptide *i*, ${\hat{y}}_{i}$ represents predicted probability of peptide *i*. Derivatives of the objective function with respect to the model parameters are calculated and used in standard backpropagation [29]. For updating weights in CNN, we used a gradient decent optimization algorithm called RMSprop, which was developed to deal with radically diminishing learning rates [30]. This algorithm converged significantly faster than conventional approaches such as Stochastic Gradient Descent (SGD), requiring only 30 epochs with the learning rate of 0.01 to reach below root mean squared error (RMSE) of 0.01 in all seven datasets we examined. After computation of each convolutional and fully connected layer, a dropout layer where a fraction (20% at each layer) of the model parameters are set to zero is applied to prevent overfitting [31]. We didn’t impose any regularization constraints as dropout already discarded a sufficient number of the parameters.

### Protein inference using CNN

The key idea behind DeepPep is that if a protein is the origin of the input peptide, the peptide probability will depend on the presence or absence of the protein from the input. To quantify this, we define the normalized change in the probability of peptide *pp*_*j*_ due to protein *p*_*i*_ as:

$$c_{i,j}=\frac{|y_{j}-\text{CNN}\left(x_{j},p_{i}\right)|}{n_{i}^{j}}$$

where CNN(*x*_*j*_, *p*_*i*_) represents the predicted probability of peptide *pp*_*j*_ in the absence of protein *p*_*i*_. The normalization quantity $n_{i}^{j}$ denotes the number of positions in protein *p*_*i*_ that were reset to zero to declare the absence of the protein. In other words, these are the number of amino acids in the protein that have a perfect match with the peptide *pp*_*j*_ and it is always a multiple of its length. This normalization is necessary as the probability difference will increase with the number of zeros that we impose. Finally, the score of protein *p*_*i*_ is assigned to be the average of *c*_*i*,*j*_ for all *j*. The scores of all proteins *p* are the outputs of DeepPep.

### Scalability of DeepPep

Given the way DeepPep represents peptide-protein matches, the memory requirement for loading inputs can be massive. For example, the input for the Yeast dataset takes 26GB. Even worse, the memory that a deep network needs is several times more than the actual input size (for storing gradients in each layer). Furthermore, in terms of CPU, computation of convolution, linear transformations and gradients in the network given such data, can pose a limit to scalability of DeepPep. However, given that any protein only matches with a handful of peptides, the input to DeepPep is largely sparse (between 95% to 99% depending on the dataset) and therefore taking advantage of this property can greatly reduce the memory and computational overhead of DeepPep (**Section 3.4**). Next we explain how to take advantage of this sparsity in the context of Deep Neural Networks.

#### Sparse representation

Given that the data is sparse, we use a sparse representation, as in **S1 Fig** (**in** S1 Text), hence only the non-sparse information is encoded along with the index of the row which contains non-sparse information for a given column (protein). This representation reduces the memory overhead approximately 98 fold.

#### Sparse calculations

Given the sparse input representation, individual layers need to preserve the sparsity across layers, hence avoiding computations involving zero records throughout. We achieve this by introducing the following:

1. Avoiding the bias term: To preserve the sparsity of data throughout layers we remove the “bias” (or offset) term in all layers. Although this has the potential of limiting the modeling capacity of the network (e.g. negative correlations), our experiments did not show a practical difference for the given datasets. This can be due to i) input dataset being non-negative, ii) peptide abundance is expected to increase monotonically with added proteins. Hence we don’t expect to have negative trends.
2. Avoiding gradient calculation: In DeepPep, only the Convolution and Linear layers have parameters (i.e. weights) for which gradient need to be calculated. When the module input is zero, there is no contribution of the back-propagated error to the local gradient, hence those calculations are omitted.

#### Shared intermediate transformations

The final phase of DeepPep consists of calculating the share of each protein in predicted peptide probabilities. In a naïve implementation, for each protein the CNN is applied on all the inputs while peptide matches for the given protein is set to zero. To make this efficient, the CNN’s output of the layer prior to the final layer is first saved without setting any match to zero. Then for each protein, only the outputs corresponding to that protein are multiplied by their related weights in the final layer to calculate the share of that protein in the final prediction. This implementation provides (*N*-1) fold improvement in terms of runtime where *N* is the number of candidate proteins.

### Comparison with other methods

We used the following four popular methods that are based on optimization, Bayesian, and constrained regression approaches to compare with DeepPep. The running time of protein inference methods including prerequisite steps of each method (e.g. DQModel, TPP pipeline) was all measured and compared on Two Intel E5-2630 v3 2.4GHz CPUs with eight cores with 64GB of RDIMM RAM.

#### Fido

Fido [32] is the Bayesian method for computing discriminative posterior protein probabilities. This method has been updated to achieve higher computational efficiency by employing probabilistic convolution trees [33]. It is based on three parameters of i) the probability of generating associated peptides from present proteins, ii) the probability of creating peptides from a noise model, and iii) the prior probability of each protein. As suggested in [34], we first ran a grid search of *α*, *β*, and *γ* using FidoChooseParameters with decoy dataset (for more information about how decoy datasets were generated, see **Section 2.2**) and executed Fido with the optimal hyper-parameters for each dataset (**S3 Table** in S1 Text). To find most accurate hyper-parameters, we set 1 for *c* parameter option in FidoChooseParameters. For Sigma49 dataset, we used the optimal hyper-parameters reported in [32], as its raw MS data was not available and thus, we weren’t able to produce a decoy dataset for hyper-parameter optimization.

#### MSBayesPro

MSBayesPro [9] utilizes the concept of peptide detectability, which is defined as the probability of detecting a peptide in a standard sample by a standard proteomics routine. We first obtain the peptide identifications and their probabilities from PeptideProphet [2]. Peptide detectabilities of a protein search database were produced using DQModel [7] and then the detectability information of the proteins present in peptide identification step are filtered in (**Section 2.2**). Finally, we run MSBayesPro to estimate the protein priors and run again with priors to obtain the final protein probabilities.

#### ProteinLasso

ProteinLasso [8] formulates the protein inference problem as a constrained Lasso regression problem, which can be solved very efficiently through a coordinate descent procedure. Similar to MSBayesPro, ProteinLasso also needs peptide detectability values as input. We adopt the same peptide detectability generation procedure used in MSBayesPro. As instructed from [8], we set *ϵ* = 0.001 and *K* = 100 in our experiment. From the peptide identification and peptide detectability files, we can calculate the *λ*_*max*_ value. Then we measure *λ*_*min*_ by assigning *ϵλ*_*max*_. *K* intervals are chosen from *λ*_*max*_ to *λ*_*min*_ on the log scale. Based on each *λ* value, ProteinLasso finally outputs a list of protein probabilities.

#### ProteinLP

ProteinLP [3] minimizes the number of proteins with non-zero probabilities under the constraint that the difference between the calculated peptide probability and the peptide probability generated from peptide identification algorithms should be less than some threshold. We set *ϵ* = 0 in the experiment for ProteinLP as instructed from [3].

### Implementation

Hyper-parameter optimization of DeepPep was performed on parallel under the CPU environment on the NCSA Blue Waters supercomputer (a petascale machine with 22,500 nodes of AMD 6276 Interlagos 2.3GHz processors, 64GB memory per node). The computing environment used for comparison of running time of protein inference methods was described in **Section 2.8**. The “Data Preparation” step is done in Python while the training and protein inference using CNN architecture are implemented with the torch7 framework. For efficient implementation of convolutional layers, sliding windows between neighboring proteins are omitted to avoid any biases coming from concatenation of strings amongst heterogeneous proteins. The source code of DeepPep and the benchmark datasets used in this study are available at https://deeppep.github.io/DeepPep/.

## Results

### Architecture optimization

We first performed empirical hyper-parameter optimization by measuring the effect of different parameters to the prediction performance. The optimal configuration (e.g. max pooling) was investigated with respect to the pooling function *pool*, number of filters, window sizes in convolution layer and pooling layer and number of nodes in fully connected layer. The performance of each configuration was evaluated using the target decoy approach on 18Mix dataset (**Section 2.2**). In this approach, the performance is measured based on how well the method differentiates target proteins from decoy proteins and therefore, this evaluation does not use the information of the true protein set. As shown in **S4 Table** (in S1 Text), DeepPep remains robust with a high AUC/AUPR value (0.94±0.009/0.93±0.008) in the spectrum of the experiments we performed (final selection is shown in Fig 2).

### Performance comparison

**Fig 3:**
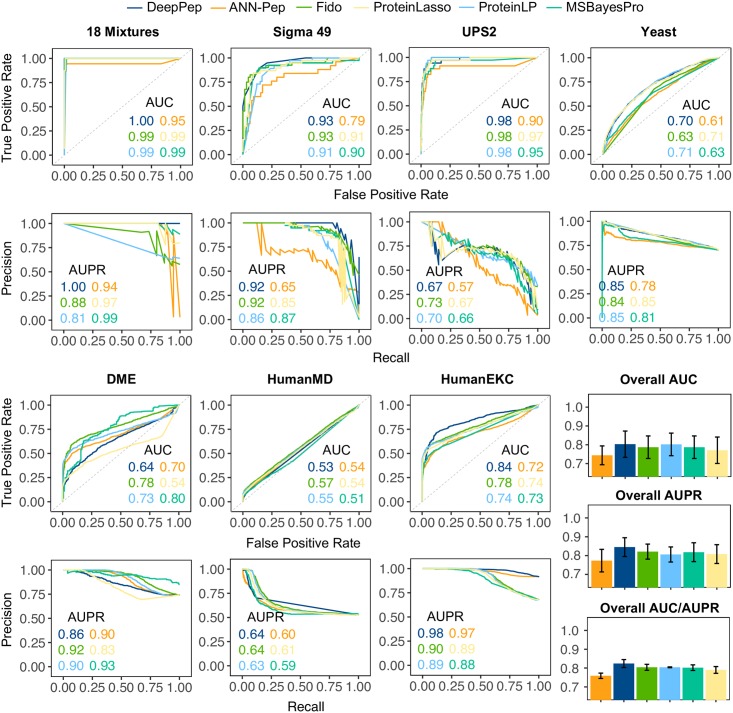
ROC (Receiver Operator Characteristic) and PR (Precision Recall) curve of DeepPep and five other methods for seven independent datasets. ANN-Pep uses the same framework of DeepPep except that its neural network architecture doesn’t employ convolution layers. Among 18 different configurations of ANN-Pep, the configuration with best performance is shown. Complete results of ANN-Pep are shown in **S5 Table** (in S1 Text).

**Fig 4:**
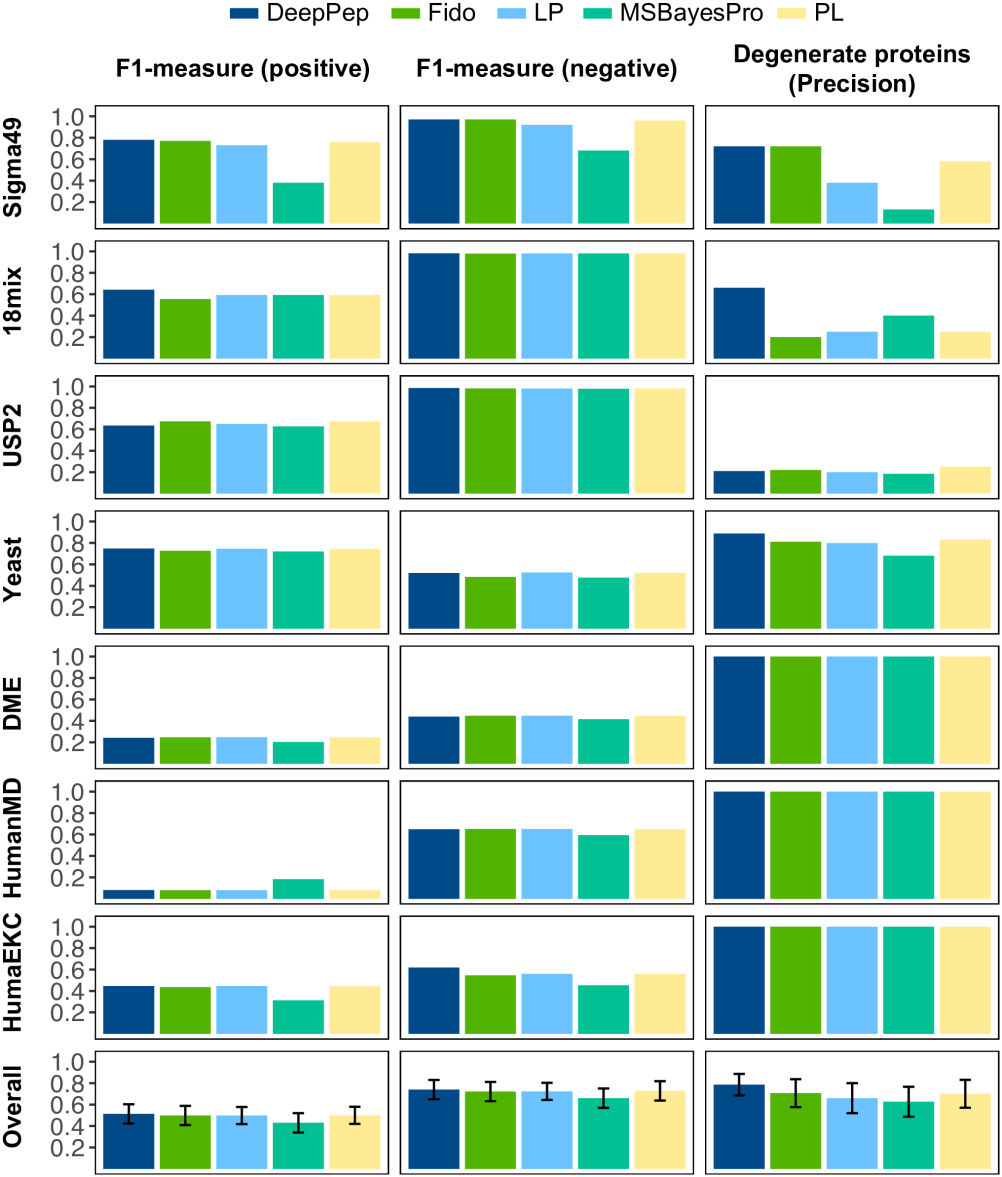
(A) F1-measure of DeepPep and four other methods for three independent sources. F1-measure was computed for positive predictions, and negative predictions. (B) Precision of degenerate proteins (proteins that have peptides with multiple protein matches) was measured for each of three datasets. That is, the proportion of degenerate proteins being known among all degenerate proteins predicted as known. The final list of inferred protein set is decided by top-k ranked proteins, where k is 38, 43, 51, 3405, 316, 282, 1316 for 18 Mixtures, Sigma49, USP2, Yeast, DME, HumanMD, and HumanEKC, respectively. As in [8], the value of *k* is determined by the number of proteins having probability of 1.0 computed by ProteinProphet [4]. LP; ProteinLP, PL; ProteinLasso.

Fig 3 depicts the performance of the six methods for the seven independent datasets with respect to ROC curve and PR curve. Overall, DeepPep shows competing performance across different datasets, ranking first by a narrow margin in overall AUC and AUPR. It is noticeable that DeepPep outperforms other methods for HumanEKC dataset. Although the AUC/AUPR performance of DeepPep are below those of other methods for DME dataset, we noticed that the performance based on the final list of inferred proteins (i.e. F1-measure) is comparable as shown in Fig 4. Furthermore, the hyper-parameters of DeepPep learned from the decoy-added 18Mix dataset might not be optimal for DME dataset, which could be improved once hyper-parameter optimization is performed separately for each of the seven datasets using the target decoy approach **Section 2.2** as done for Fido **Section 2.8.1**. DeepPep ranks first by a small margin or ties with others in first place for four (18Mix, Sigma49, Yeast and HumanEKC) out of seven datasets.

Next, we assess the degree that the convolution layers impact the performance of DeepPep. For this purpose, we compare the performance of DeepPep against traditional Artificial Neural Networks without convolution layers but otherwise similar settings (ANN-Pep). ANN-Pep uses fully connected layers to connect inputs to outputs. The results (Fig 3 and **S5 Table** in S1 Text) show that overall, DeepPep (AUC/AUPR: 0.80/0.84) outperforms ANNs with 18 different architectural configurations for seven datasets (max AUC/AUPR: 0.74/0.77), which suggests spatial dependencies within input sequences are crucial to maximize the capacity of protein inference. The performance of all 18 configuration are tabulated in **S5 Table** (in S1 Text).

We also evaluated DeepPep with other methods based on the list of predicted proteins (Fig 4). DeepPep shows comparable performance with the other four methods across different datasets with regards to positive prediction and negative prediction of inferred proteins. MSBayesPro shows top performance in positive prediction of inferred proteins for HumanMD dataset, whereas its performance is degraded on other datasets (e.g. Sigma49 and HumanEKC). Second, DeepPep is particularly sensitive in prediction of degenerate proteins, which have peptides with multiple protein matches. It is particularly notable that the performance of other methods for degenerate proteins fluctuates across the datasets of Sigma49, 18Mix, UPS2, and Yeast whereas DeepPep shows consistently competitive performance overall. Dealing with such proteins has been considered more challenging than others because there are multiple options to select protein origins of a peptide. Overall, DeepPep outperforms other methods in terms of precision for degenerate proteins.

### Visual interpretation of DeepPep

**Fig 5:**
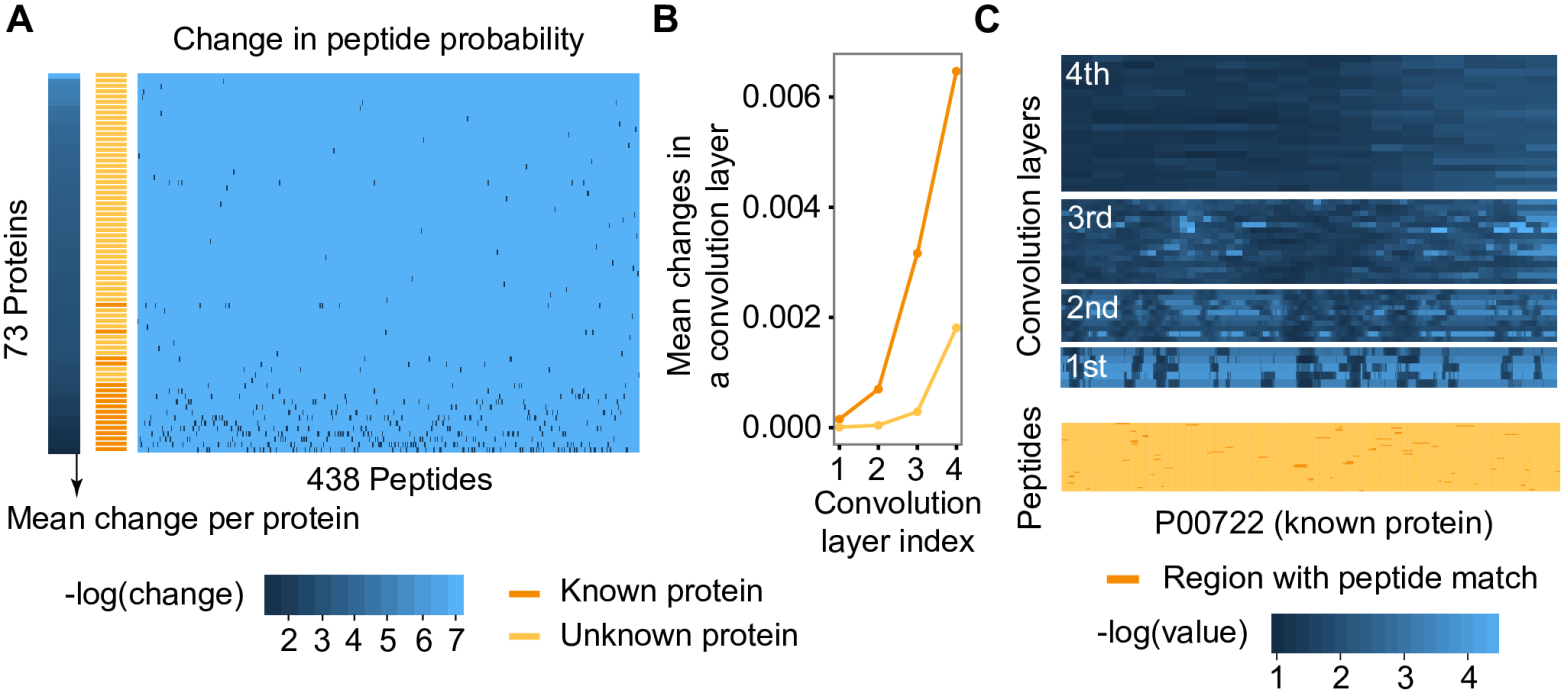
Visualization of underlying processes in DeepPep. (A) Changes in peptide probability owing to protein absence are visualized in heat map for all peptides and for all proteins in 18Mix dataset. Left bar indicates the mean changes across all peptides for each protein and it is in the decreasing order from bottom. Right bar marks known proteins in orange. (B) Mean changes of each convolution layer for known protein and unknown protein. (C) Visual representation of the averaged intermediate values for the protein P00722 in four convolutional layers for processing 68 input peptides having matches in the protein in DeepPep.

We next investigated visually underlying processes in DeepPep. As shown in Fig 5A, the mean change in peptide probability with and without a protein (left bar) highly correlates with a list of gold standard proteins (right bar). As expected, the proteins with more peptide matches undergo more changes in peptide probability in general (heat map). This is because our CNN learns the source of observed peptide probability with respect to proteome sequences and a protein with multiple peptides matches will have higher weights in the CNN than a protein with few matches. This will result in increased change in the CNN’s output when a protein that is the origin of the peptide is nullified. In the underlying processes of computing output from input in the CNN, the deeper convolution layers increase the difference between positive and negative samples (Fig 5B). One explanation is that the changes are transmitted to adjacent neurons as a pair of pooling/convolution undergoes and proteins with more peptide matches (which is likely be in the protein set) will overall impact more in neighboring neurons than proteins with few peptide matches. This interpretation is more visible in Fig 5C, which shows that in the first convolution layer, the changes are visible in regions matching with input peptides only when it is propagated to the whole segment of the protein as it gets into a deeper layer.

### Comparison of computational efficiency

We examined computational efficiency of DeepPep in comparison to other methods over seven datasets. The results (Table 1) show that the efficient implementation of DeepPep (**Section 2.7**) enables it to run between 2.5 minutes and 90 minutes depending on the size of dataset (**S2 Table** in S1 Text). We observed that MSBayesPro shows a significant delay when the dataset size becomes larger (i.e. Yeast, DME, HumanMD, and HumanEKC). Please note that the computational efficiency of Fido can be enhanced further with its advanced version (FidoCT, [33]) although its impact should be minimal in the rank as it already shows top performance overall. Apparently, although DeepPep is not the most efficient method among five methods in terms of running time of the protein inference method, we would like to point out that many other methods need prerequisite steps before execution ranging from estimation of peptide detectability (ProteinLasso and MSBayesPro) to hyper-parameter optimization using target decoy strategy (Fido), which all affect the overall running time (**S6 Table** in S1 Text). Specifically, Fido’s prerequisite step to optimize hyper-parameters based on a grid search over a decoy-added dataset necessitates to almost double the running time of TPP by adding decoy proteins on the search database, which consumes from 15 min to 29 hours more depending on the size of dataset. Considering all these hidden steps required before running actual methods, DeepPep ranks in second or third place among five methods in overall running time comparisons.

**Table 1:** Comparison of computational efficiency of five protein inference methods over six datasets. We ran three times for each method on the computer (Two Intel E5-2630 v3 2.4GHz CPUs with eight cores with 64GB of RDIMM RAM). PLP; ProteinLP, MSB; MSBayesPro, PL; ProteinLasso. HMD; HumanMD dataset, HEKC, HumanEKC dataset.

Datasets	Methods				
	PLP	MSB	PL	Fido	DeepPep
**18Mix**	1.79s (±0.05)	4.11s (±1.02)	0.23s (±0.01)	0.24s (±0.01)	150.07s (±1.07)
**UPS2**	1.81s (±0.06)	12.32s (±0.61)	3.09s (±0.18)	5.45s (±0.19)	211.68s (±1.70)
**Yeast**	742.7s (±10.78)	36120s (±190.72)	36.35s (±1.12)	3.90s (±0.24)	5421s (±113.9)
**DME**	6.34s (±0.29)	1923.36s (±12.66)	22.78s (±1.17)	0.60s (±0.07)	737.48s (±4.56)
**HMD**	150.7s (±4.54)	22640.1s (±709.17)	285.12s (±2.45)	4.55s (±0.17)	2483s (±113.84)
**HEKC**	59.09s (±3.25)	10617.8s (±222.25)	136.54s (±5.22)	3.65s (±0.22)	1152.73s (±9.80)

## Discussion

We described DeepPep, a convolutional neural network method for deep protein inference. Our results provide evidence that using sequence-level location information of a peptide in the context of proteome sequence can result in more accurate and robust protein inference. DeepPep demonstrated a competitive predictive ability (AUC of 0.80±0.18, AUPR of 0.84±0.28) in inferring proteins without need of peptide detectability on which recent methods mostly rely. This has significant implications in proteomics pipelines, where peptide detectability quantification is a major step. We also demonstrated the predictive value of the convolutional layers, by comparing DeepPep to various other ANNs, highlighting the importance of spatial dependencies in peptide/protein sequences.

In performance comparison, while DeepPep was trained on the same datasets that required protein inference, the detectability-based methods (ProteinLasso and MSBayesPro) were executed with peptide detectabilities predicted using models trained on totally different datasets. As shown in [7], detectability predictions impact the protein inference performance, therefore, training detectability prediction models on the datasets to infer the protein set might alter the reported performance of ProteinLasso and MSBayesPro at the expense of having a longer overall computation time.

The architecture of DeepPep can be extended to predict quantities of proteins beyond identification of proteins. For example, one can use the concentration or count of each peptide as an informative feature for predicting the concentration of each protein in the original sample. Furthermore, although we have addressed scalability issues in DeepPep by employing the sparsity of proteome datasets, other advances to tackle computational complexity in deep learning, for example, distributed training [35] and optimization of memory use [36], can be integrated, which will make the tool more available, anticipating the method can be deployed for most practical applications, similar to the way it was demonstrated in this work. In addition, the proposed method relies on the preceding method (PeptideProphet [2]) that identifies peptides from a given set of mass-spectra. This method has shown high precision, achieving AUC from 0.96 to 0.97 across different datasets [37]. To minimize any noise produced in identifying peptides, the proposed framework can be extended to directly take mass spectra as input (e.g. input encodes short peptide corresponding to each mass spectra and its intensity becomes desired output of CNN.).

We would like to emphasize that application of a similar architecture to the one present in DeepPep can be introduced to solve biological problems beyond protein inference. For example, it can be applied in metagenome sequencing where genetic profiles are derived directly from environmental samples and there the task will be to identify the microbial consortia [38]. Similarly, cell type inference from short RNA reads of fragmented heterogeneous cells [39] can benefit from such architecture. Doing so will add one more area where the power of deep learning can be harvested to increase prediction performance in computational biology [28, 40, 41].

## Supporting information

S1 Text Supplementary results. (DOCX)
