# Supplementary material for: DeepPep: Deep proteome inference from peptide profiles
Source: PLoS Comput Biol. 2017 Sep 5;13(9):e1005661. doi: 10.1371/journal.pcbi.1005661 (PMC5600403; doi:10.1371/journal.pcbi.1005661)
Supplement: S1 Text — (DOCX) [file pcbi.1005661.s001.docx]

| **Dataset** | **PCC** $\boldsymbol{>}$ **0.7** | **PCC** $\boldsymbol{>}$ **0.8** | **PCC** $\boldsymbol{>}$ **0.9** | **PCC** $\boldsymbol{=}$ **1.0** |
| --- | --- | --- | --- | --- |
| Sigma49 | 0.238% | 0.219% | 0.158% | 0.109% |
| 18mix | 0.005% | 0.005% | 0.004% | 0.004% |
| UPS2 | 0.037% | 0.030% | 0.029% | 0.028% |
| Yeast | 0.049% | 0.046% | 0.039% | 0.035% |
| DME | 0.006% | 0.005% | 0.002% | 0.001% |
| HumanMD | 0.006% | 0.003% | 0.001% | 0.000% |
| HumanEKC | 0.001% | 0.000% | 0.000% | 0.000% |
| **Average** | **0.050%** | **0.040%** | **0.030%** | **0.03%** |

**Table S1.** Percentage of proteins pairs having similar peptide (PCC $\boldsymbol{>}$ X) matches among all possible protein pairs.

| **Mixture name** | **# of peptides identified** | **# of unique peptides** | **# of reference proteins** | **# of proteins matching peptides** | **# of proteins in search database** | **Source [raw,db]** |
| --- | --- | --- | --- | --- | --- | --- |
| Sigma49 | 629 | 337 | 49 | 105 | 20170 | [^*^,3] |
| 18mix | 16536 | 2096 | 18 | 613 | 1818 | [4,5] |
| UPS2 | 6551 | 1982 | 48 | 789 | 20170 | [2,3] |
| Yeast | 128370 | 47670 | 4265 | 5594 | 6714 | [5,6] |
| DME | 14891 | 3474 | 2240 | 3021^&^ | 19177$\times$2 | [7,8] |
| HumanMD | 199194 | 18687 | 6106 | 11554^&^ | 20170$\times$2 | [9,3] |
| HumanEKC | 188125 | 13934 | 4575 | 6653^&^ | 20170$\times$2 | [10,3] |

**Table S2**. Summary statistics of seven datasets used for evaluation of protein inference methods. ^*^: raw data not available. ^&^: Decoy proteins are also counted.

| **Dataset** | **gamma** | **alpha** | **beta** |
| --- | --- | --- | --- |
| Sigma49^*^ | 0.9 | 0.36 | 0.05 |
| 18mix | 0.1 | 0.04 | 0.01 |
| UPS2 | 0.1 | 0.16 | 0.01 |
| Yeast | 0.9 | 0.36 | 0 |
| DME | 0.7 | 0.01 | 0 |
| HumanMD | 0.7 | 0.01 | 0 |
| HumanEKC | 0.7 | 0.04 | 0.025 |

**Table S3.** Optimal parameters of Fido estimated by a grid search using decoy dataset. ^*^: We used the optimal parameters for Sigma49 reported in [1] as its raw MS data was not available. ^&^: Decoy proteins are also counted.

| **Types of hyperparameters** | **Parameter value** | | | | | |
| --- | --- | --- | --- | --- | --- | --- |
|  | **AUC (AUPR)** | | | | | |
| Window length in conv layer | 4 | 6 | 8 | | 10 | 12 |
|  | .95 (.95) | .95 (.96) | .96 (.96) | | .95 (.95) | .95 (.94) |
| Window length in  pooling layer | 2 | 3 | 4 | | 5 | 6 |
|  | .93 (.93) | .94 (.95) | .92 (.93) | | .93 (.93) | .93 (.94) |
| # of nodes in fully connected layer | 100 | 200 | 300 | | 400 | 500 |
|  | .93 (.94) | .93 (.94) | .94 (.95) | | .95 (.95) | .96 (.96) |
| Dropout rate after each pooling layer | 0.1 | 0.2 | 0.3 | | 0.4 | 0.5 |
|  | .93 (.94) | .94 (.95) | .94 (.94) | | .91 (.91) | .91 (.90) |
| Number of conv layers | 1 | 2 | 3 | | 4 | 5 |
|  | .93 (.93) | .93 (.93) | .93 (.95) | | .95 (.96) | .92 (.93) |
| # of filters in conv layers | 5,10,15,20 | 10,20,30,40 | 15,30,45,60 | | 20,40,60,80 | 25,50,75,100 |
|  | .96 (.96) | .94 (.96) | .95 (.94) | | .93 (.94) | .93 (.93) |
| Pooling type | Max-pooling | | | Average-pooling | | |
|  | .96 (.96) | | | .96 (.95) | | |

**Table S4**. AUC and AUPR of DeepPep with different architectural configurations for decoy-added 18Mix dataset where the performance is measured based on how well the method differentiates target proteins from decoy proteins. This evaluation does not use the information of the true protein set. Default parameters are fixed with max-pooling, window length 10 in convolution layer, window length 4 in pooling layer, 500 nodes in fully connected layer, 0.2 dropout rate, (5,10,15,20) filters in convolution layers. Number in red is optimal setup with respect to the parameter under investigation. For architectures with varying number of conv layers, # of filters are fixed to 20 for each layer. conv; convolution.

| **# of layers** | **# of nodes**  **per layer** | **Dataset** | | | | | | |
| --- | --- | --- | --- | --- | --- | --- | --- | --- |
|  |  | **Sigma49** | **18mix** | **UPS2** | **Yeast** | **DME** | **HMD** | **HEKC** |
| 1 | 100 | .67 (.56) | .67 (.52) | .67 (.38) | .56 (.66) | .55 (.72) | .51 (.56) | .57 (.84) |
|  | 200 | .77 (.63) | .81 (.73) | .75 (.46) | .57 (.66) | .61 (.75) | .52 (.57) | .61 (.85) |
|  | 500 | .78 (.64) | .85 (.84) | .80 (.47) | .61 (.68) | .70 (.80) | .54 (.50) | .62 (.87) |
| 2 | 100 | .56 (.37) | .58 (.16) | .53 (.30) | .52 (.67) | .54 (.79) | .50 (.52) | .53 (.85) |
|  | 200 | .66 (.55) | .80 (.46) | .73 (.34) | .53 (.67) | .58 (.82) | .50 (.59) | .55 (.85) |
|  | 500 | .67 (.53) | .91 (.94) | .84 (.48) | .54 (.68) | .62 (.84) | .50 (.58) | .58 (.85) |
| 3 | 100 | .55 (.39) | .55 (.13) | .63 (.40) | .50 (.75) | .52 (.77) | .50 (.56) | .50 (.86) |
|  | 200 | .69 (.53) | .78 (.52) | .59 (.37) | .50 (.75) | .55 (.87) | .50 (.56) | .50 (.86) |
|  | 500 | .70 (.50) | .95 (.94) | .90 (.57) | .61 (.78) | .70 (.90) | .54 (.60) | .72 (.97) |
| 4 | 100 | .50 (.64) | .53 (.54) | .50 (.52) | .50 (.75) | .62 (.84) | .50 (.56) | .50 (.86) |
|  | 200 | .72 (.45) | .73 (.74) | .63 (.40) | .50 (.85) | .53 (.77) | .50 (.59) | .50 (.86) |
|  | 500 | .79 (.65) | .86 (.87) | .80 (.54) | .60 (.75) | .61 (.75) | .50 (.56) | .70 (.96) |
| 5 | 100 | .66 (.65) | .50 (.52) | .50 (.52) | .50 (.85) | .50 (.87) | .52 (.57) | .62 (.87) |
|  | 200 | .63 (.64) | .60 (.52) | .50 (.52) | .54 (.68) | .70 (.80) | .50 (.56) | .61 (.85) |
|  | 500 | .65 (.59) | .70 (.72) | .70 (.53) | .50 (.85) | .54 (.79) | .52 (.57) | .60 (.78) |
| 6 | 100 | .53 (.64) | .50 (.52) | .63 (.30) | .61 (.68) | .55 (.72) | .51 (.56) | .50 (.76) |
|  | 200 | .53 (.64) | .50 (.52) | .50 (.52) | .50 (.85) | .50 (.87) | .50 (.56) | .50 (.76) |
|  | 500 | .53 (.64) | .60 (.62) | .67 (.38) | .56 (.66) | .50 (.87) | .50 (.52) | .50 (.76) |

**Table S5**. AUC and AUPR (parenthesis) of ANN-Pep with different architectural configurations. HMD; HumanMD dataset, HEKC; HumanEKC dataset.

| **Datasets** | **Steps** | **Methods** | | | | |
| --- | --- | --- | --- | --- | --- | --- |
|  |  | **PLP** | **MSB** | **PL** | **Fido** | **DeepPep** |
| 18Mix | DQModel |  | 20.02$\pm$0.12 | 20.02$\pm$0.12 |  |  |
|  | TPP | 1010$\pm$42 | 1010$\pm$42 | 1010$\pm$42 | 1824$\pm$42 | 1010$\pm$42 |
|  | Opt |  |  |  | 1.75$\pm$0.01 |  |
|  | Method | 1.79$\pm$0.05 | 4.11$\pm$1.02 | 0.23$\pm$0.01 | 0.24$\pm$0.01 | 150.07$\pm$1.07 |
|  | Total | 1011$\pm$38 | 1034$\pm$39 | 1031$\pm$40 | 1826$\pm$40 | 1160$\pm$40 |
| UPS2 | DQModel |  | 405.19$\pm$0.89 | 405.19$\pm$0.89 |  |  |
|  | TPP | 3888$\pm$4 | 3888$\pm$4 | 3888$\pm$4 | 8845$\pm$347 | 3888$\pm$4 |
|  | Opt |  |  |  | 215.69$\pm$0.72 |  |
|  | Method | 1.81$\pm$0.06 | 12.32$\pm$0.61 | 3.09$\pm$0.18 | 5.45$\pm$0.19 | 211.68$\pm$1.70 |
|  | Total | 3890$\pm$3 | 4305$\pm$3 | 4296$\pm$3 | 9066$\pm$345 | 4100$\pm$3 |
| Yeast | DQModel |  | 108.31$\pm$0.68 | 108.31$\pm$0.68 |  |  |
|  | TPP | 12952$\pm$309 | 12952$\pm$309 | 12952$\pm$309 | 25459$\pm$54 | 12952$\pm$309 |
|  | Opt |  |  |  | 25.91$\pm$0.85 |  |
|  | Method | 742.7$\pm$10.78 | 36120$\pm$190 | 36.35$\pm$1.29 | 3.90$\pm$0.24 | 5421$\pm$113.9 |
|  | Total | 13695$\pm$311 | 49180$\pm$288 | 13096$\pm$304 | 25489$\pm$51 | 18373$\pm$303 |
| DME | DQModel |  | 376.01$\pm$3.06 | 376.01$\pm$3.06 |  |  |
|  | TPP | 7525$\pm$17 | 7525$\pm$17 | 7525$\pm$17 | 15380$\pm$9 | 7525$\pm$17 |
|  | Opt |  |  |  | 14.15$\pm$0.68 |  |
|  | Method | 6.34$\pm$0.29 | 1923.36$\pm$12.66 | 22.78$\pm$1.17 | 0.60$\pm$0.07 | 737.48$\pm$4.56 |
|  | Total | 7531$\pm$15 | 9824$\pm$12.34 | 7923$\pm$2.89 | 15395$\pm$8 | 8262$\pm$15 |
| HMD | DQModel |  | 405.19$\pm$0.89 | 405.19$\pm$0.89 |  |  |
|  | TPP | 103655$\pm$138 | 103655$\pm$138 | 103655$\pm$138 | 208578$\pm$301 | 103655$\pm$138 |
|  | Opt |  |  |  | 188.14$\pm$1.22 |  |
|  | Method | 150.7$\pm$4.54 | 22640.1$\pm$708 | 285.12$\pm$2.45 | 4.55$\pm$0.17 | 2483$\pm$113.84 |
|  | Total | 103805$\pm$135 | 126700$\pm$704 | 103940$\pm$136 | 208771$\pm$300 | 106138$\pm$135 |
| HEKC | DQModel |  | 405.19$\pm$0.89 | 405.19$\pm$0.89 |  |  |
|  | TPP | 36392$\pm$177 | 36392$\pm$177 | 36392$\pm$177 | 68793$\pm$5534 | 36392$\pm$177 |
|  | Opt |  |  |  | 197.65$\pm$1.67 |  |
|  | Method | 59.09$\pm$3.25 | 10617.8$\pm$222 | 136.54$\pm$5.22 | 3.65$\pm$0.22 | 1152.73$\pm$9.8 |
|  | Total | 36451$\pm$174 | 47414$\pm$209 | 36933$\pm$173 | 68994$\pm$5532 | 37544$\pm$171 |

**Table S6**. Comparison of computational efficiency of five protein inference methods including prerequisite steps over six datasets. We ran three times for each method on the computer (Two Intel E5-2630 v3 2.4GHz CPUs with eight cores with 64GB of RDIMM RAM. We omitted Sigma49 dataset in this comparison as the optimal parameter of Fido was set based on the report [1]. MSB; MSBayesPro, PL; ProteinLasso, HMD; HumanMD, HEKC; HumanEKC, DQModel; the time spent for running DQModel to estimate peptide detectability, TPP; the computation time for running TPP pipeline for a decoy dataset. Opt; the time spent for the optimization of hyper-parameters, Method; the computation time to run the method. The unit of all numbers is seconds. Note that the running time of TPP for Fido is longer than others as it requires optimizing hyper-parameters using target decoy strategy by adding decoy proteins in search database.


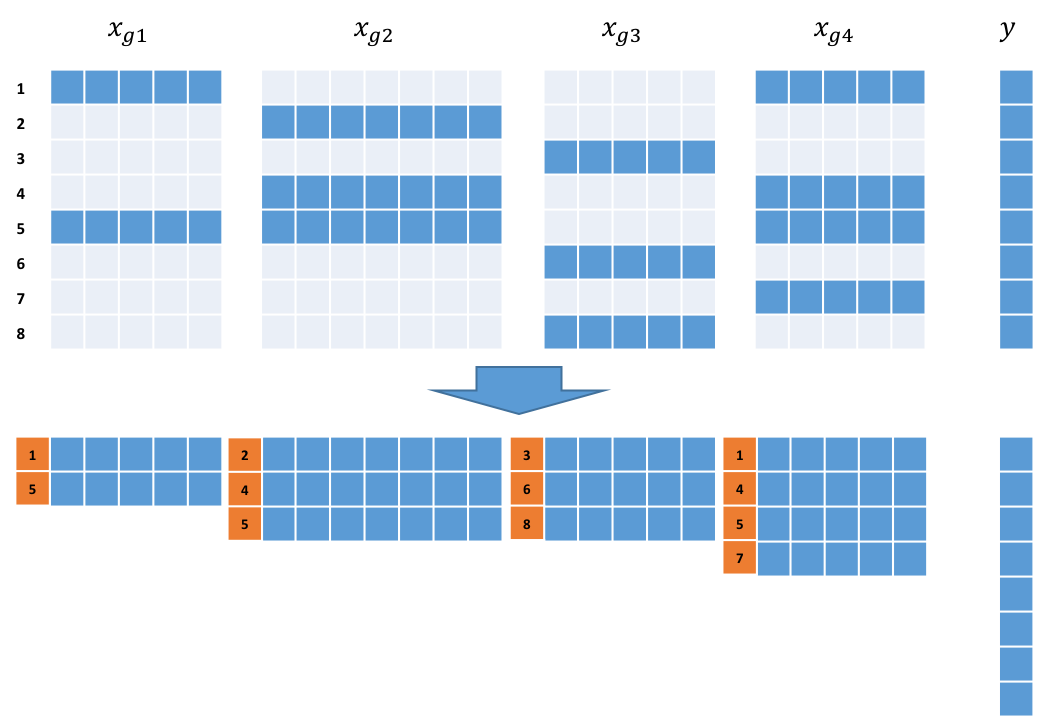


**Fig. S1.** **DeepPep** **Sparse Data Representation.** The *top section* of this figure shows an example of sparse data where blue cells represent non-sparse sections of the data (i.e. a peptide-protein match), and the rest of the cells (with light grey color) represent sparse sections (i.e. no peptide-protein match). Each row encodes protein-peptide matches for a peptide by marking it’s corresponding matches with proteins on each column. For example, $X_{g2}$ represents corresponding matches for protein 2 with peptide 2, 4 and 5. The $y$ vector represents peptide identification probabilities (for corresponding rows) which is a dense vector and remains as such. The *bottom* *section* is showing sparse representation by only encoding the non-sparse cells and using their corresponding row-id (i.e. peptide id).

**Fig. S2. Overall method comparison on seven datasets of 18Mix, Sigma49, UPS2, Yeast, DME, HumanMD, and HumanEKC.** The width of each bar represents the performance while the error bar represents the method's stability.

**References**

[1] Serang, Oliver, Michael J. MacCoss, and William Stafford Noble. "Efficient marginalization to compute protein posterior probabilities from shotgun mass spectrometry data." Journal of proteome research 9.10 (2010): 5346-5357.

[2] UPS2 RAW data. Available from: <ftp://ftp.pride.ebi.ac.uk/pride/data/archive/2013/11/PXD000331>

[3] Human protein database. Available from: <http://www.uniprot.org/uniprot/?query=reviewed%3Ayes+AND+proteome%3Aup000005640>

[4] 18MIX RAW data. Available from: <https://regis-web.systemsbiology.net/PublicDatasets/18_Mix/Mix_2/LTQ/Mix_2_LTQ_mzXML.tar.gz>

[5] 18MIX plus contaminants protein database. Available from: <https://regis-web.systemsbiology.net/PublicDatasets/database/18mix_db_plus_contaminants_20081209.fasta>

[5] Yeast RAW data. Available from: <http://www.marcottelab.org/MSdata/Data_02/DATA/>

[6] Yeast protein database. Available from: <http://www.marcottelab.org/MSdata/FASTA/sc_SGD_0604.fasta>

[7] DME RAW data. Available from: <ftp://ftp.peptideatlas.org/pub/PeptideAtlas/Repository/PAe001349>

[8] DME protein database. Available from: ftp://ftp.peptideatlas.org/pub/PeptideAtlas/Repository/PAe001349/PAe001349_4262_searchdatabase_sequest.fasta

[9] HumanMD RAW data. Available from: <http://www.marcottelab.org/MSdata/Data_05/DATA/RAW/>

[10] HumanEKC RAW data. Available from: <http://www.marcottelab.org/MSdata/Data_07/DATA/REFERENCE/>
